# Consumption of meals prepared away from home is associated with inadequacy of dietary fiber, vitamin C and mineral intake among Japanese adults: analysis from the 2015 National Health and Nutrition Survey

**DOI:** 10.1186/s12937-021-00693-6

**Published:** 2021-04-23

**Authors:** Mai Matsumoto, Aki Saito, Chika Okada, Emiko Okada, Ryoko Tajima, Hidemi Takimoto

**Affiliations:** Department of Nutritional Epidemiology and Shokuiku, National Institutes of Biomedical Innovation, Health, and Nutrition, 1-23-1 Toyama, Shinjuku-ku, Tokyo, 162-8636 Japan

**Keywords:** Meal prepared away from home, Nutrition inadequacy, Mineral, Japanese, NHNS

## Abstract

**Background:**

Consumption of home-prepared meals may lead to better nutritional intake. Few studies have examined the association between the frequency of consuming meals prepared away from home and the inadequacy of nutritional intake based on nutritional standards and food group intake. We therefore aimed to investigate this issue among Japanese adults.

**Methods:**

This study was a secondary analysis of the 2015 National Health and Nutrition Survey in Japan, involving 921 men and 1300 women aged 18–64 years, conducted as a cross-sectional survey. The frequency of consuming meals prepared away from home was defined using two questions inquiring about the frequency of eating out and take-away meals. Data on dietary intake were collected using a one-day semi-weighed household dietary record. Participants were stratified into three groups based on the frequency of consuming meals prepared away from home (High, Moderate, Low). The inadequacy of each nutrient intake was assessed by comparing estimated average requirement (EAR) level for 14 nutrients and the range of the dietary goal (DG) for seven nutrients according to the 2015 version of the Dietary Reference Intakes for Japanese. Group differences in nutrients adequacy were assessed using the covariate and logistic regression analysis. Food intake was also compared across the groups by classifying each food item into 17 groups based on Standard Tables of Food Composition.

**Results:**

The proportion of participants who consumed home-prepared meals almost every day were 34.9% among men and 46.8% among women, and the proportion of those consuming a higher frequency of meals prepared away from home were 14.7 and 6.3%, respectively. A higher frequency of consuming meals prepared away from home was associated with inadequacy of dietary fiber, vitamin C and minerals (iron, magnesium and potassium) intake, and with lower intake of vegetables and higher intake of fat and oils.

**Conclusions:**

High frequency of consuming meals prepared away from home was associated with insufficient intake of dietary fiber, vitamin C and multiple minerals among Japanese adults. These nutrients may be the potential target of interventions aimed at improving nutrient intake in individuals who predominantly eat food prepared away from home.

**Supplementary Information:**

The online version contains supplementary material available at 10.1186/s12937-021-00693-6.

## Background

The frequency of consuming meals prepared away from home has been reported to influence the quality of dietary intake [1]. Consuming meals prepared away from home is associated with higher intake of energy, fat, and sodium, and with lower intake of dietary fiber, vitamin C, and several minerals such as iron and calcium [2–13]. Additionally, eating meals prepared away from home has been linked to reduced consumption of healthy foods such as vegetables, fruit and dairy products [14–18]. Moreover, increased frequency of eating out and take-away meals has been associated with an increase in body weight, body mass index (BMI), and waist circumference [14, 18–20], and increased risk of obesity [21–23], insulin resistance, diabetes mellitus [16, 23, 24], and depression [9, 25, 26]. These findings suggest that a higher frequency of eating meals prepared away from home can affect not only the quality of diet, but also physical health.

A shift from food prepared at home to convenient/easy-to-prepare food and food prepared outside the home has been reported in the United States [27]. Additionally, high frequency of consumption of food prepared away from home has been reported in other high-income countries such as United Kingdom, and Japan [18, 28]. As the frequency of consumption of food prepared away from home increases, studies have examined the association between the frequency of consuming meals prepared away from home and intakes of energy and specific nutrients such as fat, sodium, vitamin C, iron, and calcium [2–13]. However, there are only a small number of studies that compared the nutritional adequacy of the subjects’ dietary intakes, according to their frequency of consuming meals prepared away from home [18]. Thus, it is crucial to examine the association between frequency of consuming meals prepared away from home and overall dietary quality in order to identify more effective public health nutritional intervention. In addition, it would be helpful to assess food group intake according to the frequency of meals prepared away from home in order to understand its relationship with the nutrient intake adequacy. Therefore, we aimed to examine the association of the frequency of consuming meals prepared away from home with nutrient intake inadequacy and food group intake among Japanese adults aged 18–64 years using data from the 2015 National Health and Nutrition Survey in Japan (NHNS).

## Methods

### Data source and study population

The NHNS is a nationally representative cross-sectional annual survey conducted by local public health centers under the supervision of the Ministry of Health, Labour, and Welfare. The present study was based on data from the 2015 NHNS conducted between November 1 to 30, 2015. Details of the 2015 NHNS has been described elsewhere [29, 30]. Briefly, the participants, who included households and family members (aged > 1 year as of November 1, 2015) in 300 areas, were stratified and randomly extracted from the general census areas in the Comprehensive Survey of Living Conditions in 2015. The 2015 NHNS consisted of physical examination, dietary survey, and lifestyle questionnaire. A total of 3507 out of 5327 eligible households (65.8%) and 8583 people participated in the survey. This current study included 5048 adults aged 18–64 years. We excluded participants with missing data required for analysis in the present study, such as dietary information (*n* = 1127), body weight (*n* = 592), smoking status or/and habitual alcohol consumption (*n* = 270). Moreover, we excluded those with missing data on the frequency of eating out and take-away meals (*n* = 5). We also excluded those who skipped breakfast, lunch, or/and dinner (*n* = 749), because meal skipping may affect nutrient and food intakes [31, 32], and lactating or pregnant women who may have changed their usual dietary habits (*n* = 84) [33]. Thus, the final participants consisted of 2221 Japanese adults aged 18–64 years (921 men and 1300 women).

The permission to use the 2015 NHNS data was obtained from the Ministry of Health, Labour, and Welfare, and only anonymised information was availed for this study. As this survey was conducted according to the Health Promotion Act, all participants gave informed consent to the local government, and approval from Institutional Review Board was not required.

### Dietary assessment

Dietary intake data was collected using a one-day semi-weighed household dietary record administered in November 2015, excluding Sundays and public holidays. Prior to completing the survey, trained fieldworkers (mainly registered dieticians) provided an outline of the survey and explained to the participants how to complete the dietary record. The main record-keepers in the household (members who are usually responsible for preparing meals) were instructed to weigh all foods and beverages consumed by the household members and the amount of food waste and leftovers and record their names and weights on recording forms. Additionally, the main record-keepers recorded the approximate proportions of the food consumed by each household member when members shared foods from the same dish to enable estimation of individual intake. If weighing was not possible because the meal was consumed away from the home, the portion size consumed, or quantity of foods and details of any leftovers was estimated. Also, participants reported the type of meals consumed at breakfast, lunch, and dinner on the recording day according to the following categories: prepared at home, take-away meals (dishes prepared outside home, but eaten at home), eating out at a restaurant and a fast-food store, or other meals prepared outside the home (food served at nursery school, kindergarten, elementary school, junior high school, high school, or workplace). This selection was identified by the main dishes (staple food in case there were no main side dishes).

Trained fieldworkers visited each household and checked for any missing information and errors. In accordance with the survey manual of the NHNS, the trained fieldworkers converted these estimates of portion sizes or quantity of foods into weights of foods and coded each food item, according to the NHNS food number lists based on the Standard Tables of Food Composition in Japan [34] to calculate the intake of energy and nutrients. The trained fieldworkers inputted collected dietary intake data using software specifically developed for the NHNS.

Energy and nutrients were calculated based on the 2010 Standard Tables of Food Composition in Japan, and food items were classified into 17 groups based on the definition of the Standard Tables of Food Composition [34]. In this study, we adjusted the observed dietary intake for energy requirement to minimize errors associated with self-reporting assessment, using the density method. To render the comparison between the reported nutrient intake and the Dietary Reference Intake for Japanese (DRIs) values [35] practically possible, the following calculation was used: energy-adjusted intake (units/day) = observed intake (units/day) × estimated energy requirement (EER) (kcal/day)/observed energy intake (kcal/day). EER for each participant was assumed as when their physical activity level was at the second level in the Japanese DRIs (PAL = 1.75). For protein, total fat, saturated fat, and carbohydrate, percentage of daily energy intake using reported values (crude) for each macronutrient was also calculated. Additionally, food intake values were energy-adjusted using the density method (i.e. their amounts per EER for food groups: energy-adjusted intake (g/day) = observed intake (g/day) × EER (kcal/day)/observed energy intake (kcal/day)).

### Frequency of consuming meals prepared away from home

**Fig. 1:**
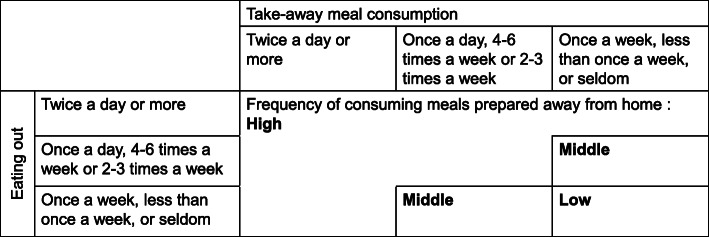
Classification of study participants based on frequency of consuming meals prepared away from home

The frequency of consuming meals prepared away from home was assessed by the combination of two questions in the lifestyle questionnaire asking about the frequency of eating out and take-away meals. Participants reported the frequency of eating out and take-away meals (twice a day or more, once a day, 4–6 times per week, 2–3 times per week, once a week, less than once a week, seldom). Figure 1 shows the classification of participants into three groups according to the frequency of consuming meals prepared away from home, based on the previous reports [3, 15, 18]. Participants who answered, “twice a day or more” to either question and those who answered, “once a day,” “4–6 times a week” or “2–3 times a week” to both questions were classified into the High group (high frequency of consuming meals prepared away from home). Participants who responded to both questions “once a week,” “less than once a week,” “seldom” were classified in the Low group (low frequency of consuming meals prepared away from home). If none of the above applies to those, participants were classified into the Moderate group.

### Determination of inadequate nutrient intake

Inadequate intake of each nutrient was determined by comparing energy-adjusted nutrient levels with the relevant dietary reference value according to the Japanese DRIs, using a previously reported method [36–38]. In the Japanese DRIs, different types of dietary reference values were established according to their purpose. The estimated average requirement (EAR) is set to prevent insufficient intake of nutrients, whereas the tentative dietary goal (DG) to prevent lifestyle-related diseases is set to prevent non-communicable diseases.

Nutrient intake inadequacy was defined as follows: energy-adjusted intake level below EAR was considered as inadequate using the cut-point method for the following 14 nutrients with known EARs: protein, vitamin A (as retinol activity equivalents), vitamin B_1_, vitamin B_2_, niacin (as niacin equivalent), vitamin B_6_, vitamin B_12_, folate, vitamin C, calcium, magnesium, iron, zinc, and copper. Regarding iron intake in menstruating women, we applied the value < 9.3 mg/day as recommended by the World Health Organization (WHO) (bioavailability of iron as 15%, probability of inadequacy as 50%) [39] for women aged 20–49 years because the cut-point method is less applicable to these populations [40, 41]. For the following seven nutrients, the intake level (energy-adjusted intake level for total dietary fiber, sodium (as salt-equivalent) and potassium) outside the range of DG values was considered as inadequate: protein (as % energy: 13–20%), total fat (as % energy: 20–30%), saturated fat (as % energy: 7% or less), carbohydrate (as % energy: 50–65%), total dietary fiber (man; 20 g/day or more, woman; 18 g/day or more), sodium (as salt-equivalent: man; less than 8.0 g/day, woman; less than 7.0 g/day), and potassium (man; 3000 mg/day or more, woman; 2600 mg/day or more).

### Other variables

Body height (to the nearest 0.1 cm) and weight (to the nearest 0.1 kg) were measured for approximately 90% of the participants by trained field workers according to standardised procedures. For the remaining participants, height and weight were measured either by other household members at home or were self-reported. BMI was calculated as weight (kg) divided by height (m) squared. Smoking status and alcohol drinking habits during the preceding month were assessed by a self-administered questionnaire.

### Statistical analysis

All statistical analyses were stratified by sex. The differences in characteristics among three groups according to the frequency of consuming meals prepared away from home were compared using the chi-square test for categorical variables and analysis of variance (ANOVA) for continuous variables. Differences in daily energy-adjusted nutrients and food group intake among the three groups according to the frequency of consuming meals prepared away from home were assessed by ANOVA in the crude model and a covariate analysis (ANCOVA) in the adjusted model. Dunnett test, with the Low group as reference, was performed in the post-hoc test. The nutritional inadequacy of each nutrient intake was represented as the proportion of participants whose energy-adjusted intake was below the EAR or outside the range of the DG in each group. Logistic regression analysis was used to examine the difference in the prevalence of meeting DRIs based on the High and Moderate groups according to the frequency of consuming meals prepared away from home compared with the Low group in the crude and adjusted model. Confounding factors considered in the adjusted model were age category (18–34, 35–50, and 51–64 years), occupation (professional/manager, sales/service/clerical, security/transportation/labour, student, housekeeper, and not in paid employment), living alone or not (yes or no), region (Hokkaido/Tohoku, Kanto, Hokuriku/Tokai, Kinki, Shikoku/Chugoku, Kyusyu), current smoker (yes or no) and habitual alcohol drinker (yes or no), which was reported as a factor affecting the frequency of consuming meals prepared away from home [8, 42]. All statistical analyses were performed with SAS statistical software, version 9.4 (SAS Institute Inc., Cary, NC, USA). All reported *P* values were two-tailed, with a *P*-value < 0.05 considered statistically significant.

## Results

Table 1 shows the basic characteristics of participants according to their frequency of consuming meals prepared away from home. The proportion of participants classified into the High, Moderate, and Low groups were 14.7, 50.5, and 34.9% for men, and 6.3, 46.9, and 46.8% for women, respectively. The mean age in the Low group was significantly higher than other groups (*p* = 0.001 in men and *p* = 0.004 in women). Additionally, there were more men and women living alone in the High group (*p* < 0.001). The residing region significantly differed in women, with more women in the High group living in the urban Kanto area (*p* = 0.002). In the High group, approximately 90% of men and 70% of women reported eating out or having take-away meals in their one-day dietary records as well, which showed a significantly higher proportion compared to other groups (*p* < 0.001). There were no differences in mean BMI, type of occupation, current smoking and consumption of snacks across the groups in both men and women.

**Table 1:** Characteristics of 2221 Japanese adults aged 18–64 years according to their frequency of consuming meals prepared away from home [Mean (SD) or n (%)]

	Men							Women						
	Frequency of consuming meals prepared away from home						p^*^	Frequency of consuming meals prepared away from home						p^*^
	Low (*n* = 321)		Moderate (*n* = 465)		High (*n* = 135)			Low (*n* = 608)		Moderate (*n* = 610)		High (*n* = 82)		
Age (years), Mean (SD)	49.6	(11.8)	47.5	(12.4)	44.8	(12.1)	**0.001**	48.8	(11.0)	47.5	(11.7)	44.7	(11.4)	**0.004**
Age category, n (%)							**0.005**							**0.016**
18–34 years	44	(13.7)	82	(17.6)	33	(24.4)		67	(11.0)	95	(15.6)	16	(19.5)	
35-50 years	103	(32.1)	173	(37.2)	52	(38.5)		244	(40.1)	241	(39.5)	39	(47.6)	
51–64 years	174	(54.2)	210	(45.2)	50	(37.0)		297	(48.9)	274	(44.9)	27	(32.9)	
Body mass index (kg/m^2^), Mean (SD)	23.8	(3.4)	23.8	(3.5)	24.5	(4.4)	0.132	22.0	(3.4)	22.1	(3.7)	22.0	(3.8)	0.909
Body mass index category, n (%)							0.269							0.190
Underweight (< 18.5)	6	(1.9)	19	(4.1)	5	(3.7)		59	(9.7)	80	(13.1)	13	(15.9)	
Normal (18.5–25)	202	(62.9)	302	(65.0)	80	(59.3)		449	(73.9)	424	(69.5)	53	(64.6)	
Overweight (25≤)	113	(35.2)	144	(31.0)	50	(37.0)		100	(16.5)	106	(17.4)	16	(19.5)	
Occupation, n (%)							0.104							0.097
Professional / manager	105	(32.7)	162	(34.8)	55	(40.7)		99	(16.3)	84	(13.8)	18	(22.0)	
Sales / service / clerical	65	(20.3)	117	(25.2)	36	(26.7)		232	(38.2)	263	(43.1)	38	(46.3)	
Security / transportation / labour	119	(37.1)	135	(29.0)	29	(21.5)		66	(10.9)	45	(7.4)	6	(7.3)	
Student	3	(0.9)	10	(2.2)	4	(3.0)		7	(1.2)	14	(2.3)	2	(2.4)	
Housekeeper	3	(0.9)	6	(1.3)	2	(1.5)		193	(31.7)	194	(31.8)	17	(20.7)	
Not in paid employment	26	(8.1)	35	(7.5)	9	(6.7)		11	(1.8)	10	(1.6)	1	(1.2)	
Living alone, n (%)	11	(3.4)	44	(9.5)	22	(16.3)	**< 0.001**	39	(6.4)	41	(6.7)	14	(17.1)	**0.002**
Region, n (%)							0.093							**0.002**
Hokkaido and Tohoku	41	(12.8)	47	(10.1)	15	(11.1)		63	(10.4)	56	(9.2)	13	(15.9)	
Kanto	89	(27.7)	176	(37.9)	42	(31.1)		172	(28.3)	207	(33.9)	36	(43.9)	
Hokuriku and Tokai	60	(18.7)	81	(17.4)	21	(15.6)		101	(16.6)	132	(21.6)	15	(18.3)	
Kinki	53	(16.5)	82	(17.6)	31	(23.0)		126	(20.7)	100	(16.4)	10	(12.2)	
Shikoku and Chugoku	39	(12.2)	34	(7.3)	11	(8.2)		67	(11.0)	54	(8.9)	3	(3.7)	
Kyusyu	39	(12.2)	45	(9.7)	15	(11.1)		79	(13.0)	61	(10.0)	5	(6.1)	
Current smoker, n (%)	102	(31.8)	140	(30.1)	54	(40.0)	0.094	47	(7.7)	55	(9.0)	7	(8.5)	0.720
Habitual alcohol drinker, n (%)	117	(36.5)	172	(37.0)	45	(33.3)	0.736	49	(8.1)	77	(12.6)	10	(12.2)	**0.029**
Frequency of consuming meals prepared away from home on the dietary recording day							**< 0.001**							**< 0.001**
2 times or more, n (%)	18	(5.6)	67	(14.4)	52	(38.5)		14	(2.3)	49	(8.0)	15	(18.3)	
Once, n (%)	106	(33.0)	219	(47.1)	68	(50.4)		128	(21.1)	233	(38.2)	41	(50.0)	
None, n (%)	197	(61.4)	179	(38.5)	15	(11.1)		466	(76.6)	328	(53.8)	26	(31.7)	
Consumption of snacks on the dietary recording day, n (%)	203	(63.2)	293	(63.0)	82	(60.7)	0.870	476	(78.3)	460	(75.4)	55	(67.1)	0.066

*SD* standard deviation

* Means for continuous values were compared by an analysis of variance and proportions for categorical values were compared by the chi-square test between High, Moderate and Low groups

Table 2 shows the energy-adjusted nutrient intakes on the dietary recording day according to the frequency of consuming meals prepared away from home. Among men, the energy-adjusted intake of protein, calcium, iron, copper, dietary fiber and potassium was significantly lower in the High group than in the Low group (*p* = 0.020, 0.044, 0.008, 0.027, 0.002 and 0.004, respectively). In women, the energy-adjusted intake of folate, calcium, magnesium, iron and potassium in the Moderate group was significantly lower than in the Low group (*p* = 0.046, 0.036, 0.014, 0.001 and 0.026, respectively). Energy-adjusted dietary fiber intake was higher in the Low group compared the Moderate and High groups (*p* = 0.005).

**Table 2:** Energy-adjusted nutrient intakes on the dietary recording day among 2221 Japanese adults aged 18–64 years according to frequency of consuming meals prepared away from home [Mean (SD)]†

	Men								Women							
	Frequency of consuming meals prepared away from home						P^ǁ^	P^¶^	Frequency of consuming meals prepared away from home						P^ǁ^	P^¶^
	Low (*n =* 321)		Moderate (*n =* 465)		High (*n =* 135)				Low (*n =* 608)		Moderate (*n =* 610)		High (*n =* 82)			
Energy, kcal/d	2314	(592)	2302	(571)	2241	(554)	0.454	0.282	1771	(430)	1785	(423)	1799	(499)	0.771	0.710
Nutrients with EAR																
Protein, g/d	91	(18)	91	(17)	87	(19)^*^	**0.031**	**0.020**	73	(14)	73	(13)	72	(15)	0.598	0.782
Vitamins																
Vitamin A, μg RE/d	614	(727)	662	(751)	527	(366)	0.136	0.181	617	(910)	559	(424)	469	(248)	0.111	0.084
Vitamin B_1_, mg/d	1.1	(0.41)	1.1	(0.42)	1.1	(0.44)	0.455	0.319	0.9	(0.31)	0.90	(0.30)	0.93	(0.34)	0.653	0.646
Vitamin B_2_, mg/d	1.4	(0.46)	1.4	(0.48)	1.4	(0.51)	0.524	0.453	1.3	(0.46)	1.2	(0.39)	1.3	(0.48)	0.290	0.388
Niacin, mg/d	35	(9.8)	36	(11.2)	33	(10.5)	0.058	0.075	28	(7.6)	28	(7.5)	28	(8.5)	0.987	0.964
Vitamin B_6_, mg/d	1.4	(0.42)	1.4	(0.42)	1.3	(0.69)	0.135	0.291	1.2	(0.38)	1.2	(0.37)	1.1	(0.37)	0.569	0.724
Vitamin B_12_, μg/d	7.7	(7.7)	7.8	(7.7)	6.5	(6.2)	0.184	0.333	6.0	(6.1)	5.8	(6.1)	6.2	(5.5)	0.756	0.612
Folate, μg/d	352	(135)	363	(153)	334	(148)	0.120	0.225	335	(144)	319	(123)^*^	307	(119)	**0.044**	**0.046**
Vitamin C, mg/d	103	(70)	103	(66)	87	(61)	**0.032**	0.162	110	(71)	103	(69)	92	(71)	**0.040**	0.128
Minerals																
Calcium, mg/d	580	(258)	578	(263)	519	(245)^*^	**0.045**	**0.044**	561	(230)	525	(223)^*^	523	(243)	**0.019**	**0.036**
Magnesium, mg/d	315	(93)	315	(88)	294	(89)	**0.046**	0.084	271	(75)	260	(71)^*^	256	(74)	**0.011**	**0.014**
Iron, mg/d	9.5	(2.9)	9.7	(2.8)	8.9	(2.6)^*^	**0.007**	**0.008**	8.6	(2.8)	8.0	(2.2)^*^	7.9	(2.5)	**< 0.001**	**0.001**
Zinc, mg/d	10.7	(2.2)	10.8	(2.5)	10.5	(2.5)	0.635	0.398	8.4	(1.8)	8.4	(1.8)	8.2	(1.9)	0.600	0.684
Copper, mg/d	1.5	(0.32)	1.5	(0.34)	1.4	(0.38)^*^	**0.029**	**0.027**	1.2	(0.29)	1.2	(0.29)	1.2	(0.34)	0.061	0.060
Nutrients with DG																
Protein, %energy	14.3	(2.9)	14.3	(2.6)	13.5	(3.0)^*^	**0.005**	**0.015**	15.0	(3.0)	14.9	(2.8)	14.6	(3.0)	0.451	0.800
Fat, %energy	25.1	(6.7)	26.3	(6.9)	26.9	(6.8)	**0.020**	0.141	28.0	(7.1)	28.8	(7.2)	28.5	(7.3)	0.154	0.166
Saturated fat, %energy	6.6	(2.4)	6.8	(2.4)	7.0	(2.7)	0.212	0.673	7.6	(2.6)	7.9	(2.8)	7.8	(2.8)	0.165	0.129
Carbohydrate, %energy	60.5	(8.0)	59.5	(8.1)	59.7	(7.2)	0.173	0.283	57.0	(8.1)	56.3	(8.1)	56.8	(8.4)	0.349	0.322
Dietary fiber, g/day	18.1	(6.8)	17.9	(6.6)	15.9	(5.5)^*^	**0.002**	**0.002**	16.5	(6.0)	15.6	(6.0)^*^	14.8	(5.1)^*^	**0.004**	**0.005**
Sodium (salt-equivalent), day	13.0	(4.1)	13.1	(4.2)	13.4	(3.9)	0.603	0.760	10.3	(3.4)	10.3	(3.1)	10.6	(3.9)	0.811	0.906
Potassium, mg/d	2831	(832)	2799	(801)	2547	(908)^*^	**0.003**	**0.004**	2545	(739)	2444	(704)^*^	2372	(705)	**0.016**	**0.026**

*DG* tentative dietary goal for preventing lifestyle-related disease, *EAR* estimated average requirement, *SD* standard deviation

^†^ Nutrients expressed as amount per day were energy-adjusted by using the following equation: energy-adjusted intake (units/day) = observed dietary intake (units/day) × estimated energy requirement (kcal/day)/observed energy intake (kcal/day)

^‡^ Sum of retinol, β-carotene/12, α-carotene/24, and cryptoxanthin/24

^§^ Sum of niacin and protein/6000

^ǁ^ The *p* values are shown for an analysis of variance to analyze differences of nutrient intake between three groups

^¶^ The *p* values are shown for covariate analysis to analyze difference of nutrient intake between three groups adjusted for confounding variables of age category (18–34, 35–50, and 51–64 years), occupation (professional / manager, sales / service / clerical, security / transportation / labour, student, housekeeper, and not in paid employment), living alone (yes or no), region (Hokkaido and Tohoku, Kanto, Hokuriku and Tokai, Kinki, Shikoku and Chugoku and Kyusyu), current smoker (yes or no) and habitual alcohol drinker (yes or no). ^*^ There is significant difference by Dunnett test compared with Low group in the adjusted model

The multivariate-adjusted odds ratios (ORs) for nutrient intake inadequacy according to the frequency of consuming meals prepared away from home are shown in Table 3. Most men and women had adequate intake of protein, niacin and copper in comparison to EAR. Among men, the proportion of those having inadequate intake of iron, protein %energy, dietary fiber, and potassium in the High group was significantly higher than in the Low group. The multivariate-adjusted ORs [95% confidence interval (CI)] for inadequacy of iron, protein %energy, dietary fiber, and potassium in the High group compared with the Low group (reference) were 2.03 [1.03–4.01], 1.58 [1.03–2.40], 1.91 [1.17–3.12], and 2.17 [1.33–3.55], respectively. Women in the High group were less likely to have adequate vitamin C intake compared to women in the Low group (OR [95% CI]; 1.72 [1.05–2.80]). For magnesium and dietary fiber, the multivariate-adjusted ORs were significantly higher in the Moderate group than in the Low group (ORs [95% CI); 1.31 [1.03–1.65] and 1.32 [1.03–1.69]).

**Table 3:** Multivariate-adjusted ORs for the presence of inadequate nutrient intake (based on EAR and DG) compared with the meeting EAR and DG for frequency of consuming meals prepared away from home among 2221 Japanese adults aged 18–64 years

	Men						Women					
	Frequency of consuming meals prepared away from home						Frequency of consuming meals prepared away from home					
	Low (*n =* 321)		Moderate (*n =* 465)		High (*n =* 135)		Low (*n =* 608)		Moderate (*n =* 610)		High (*n =* 82)	
Nutrients with EAR												
Protein, *n*, %	3	0.9	2	0.4	0	0	3	0.2	0	0	0	0
Crude OR (95% CI)	–		–		–		–		–		–	
Adjusted OR (95% CI)	–		–		–		–		–		–	
Vitamins												
Vitamin A, *n*, %	216	67.3	289	62.2	100	74.1	325	53.5	325	53.3	48	58.5
Crude OR (95% CI)	1.00 (Reference)		0.80 (0.59–1.08)		1.39 (0.89–2.18)		1.00 (Reference)		0.99 (0.79–1.24)		1.23 (0.77–1.96)	
Adjusted OR (95% CI)	1.00 (Reference)		0.81 (0.60–1.10)		1.41 (0.88–2.24)		1.00 (Reference)		0.97 (0.77–1.22)		1.18 (0.73–1.90)	
Vitamin B_1_, *n*, %	197	61.4	288	61.9	96	71.1	362	59.5	362	59.3	43	52.4
Crude OR (95% CI)	1.00 (Reference)		1.02 (0.76–1.37)		1.55 (1.00–2.39)		1.00 (Reference)		0.99 (0.80–1.25)		0.75 (0.47–1.19)	
Adjusted OR (95% CI)	1.00 (Reference)		1.00 (0.74–1.34)		1.44 (0.92–2.25)		1.00 (Reference)		1.01 (0.80–1.27)		0.77 (0.48–1.23)	
Vitamin B_2,_ *n*, %	136	42.4	182	39.1	70	51.9	178	29.3	173	28.4	25	30.5
Crude OR (95% CI)	1.00 (Reference)		0.88 (0.66–1.17)		1.47 (0.98–2.19)		1.00 (Reference)		0.96 (0.75–1.23)		1.06 (0.64–1.75)	
Adjusted OR (95% CI)	1.00 (Reference)		0.86 (0.64–1.16)		1.46 (0.96–2.22)		1.00 (Reference)		0.92 (0.71–1.18)		1.00 (0.60–1.66)	
Niacin, *n*, %	1	0.3	0	0	0	0	0	0	0	0	0	0
Crude OR (95% CI)	–		–		–		–		–		–	
Adjusted OR (95% CI)	–		–		–		–		–		–	
Vitamin B_6,_ *n*, %	107	33.3	154	33.1	59	43.7	212	34.9	221	36.2	32	39.0
Crude OR (95% CI)	1.00 (Reference)		0.99 (0.73–1.34)		**1.55 (1.03–2.34)**		1.00 (Reference)		1.06 (0.84–1.34)		1.20 (0.74–1.92)	
Adjusted OR (95% CI)	1.00 (Reference)		0.95 (0.70–1.30)		1.36 (0.88–2.09)		1.00 (Reference)		1.04 (0.82–1.32)		1.17 (0.72–1.90)	
Vitamin B_12,_ *n*, %	46	14.3	53	11.4	18	13.3	138	22.7	123	20.2	17	20.7
Crude OR (95% CI)	1.00 (Reference)		0.77 (0.50–1.17)		0.92 (0.51–1.65)		1.00 (Reference)		0.86 (0.65–1.13)		0.89 (0.51–1.57)	
Adjusted OR (95% CI)	1.00 (Reference)		0.75 (0.49–1.15)		0.85 (0.46–1.56)		1.00 (Reference)		0.82 (0.62–1.07)		0.76 (0.43–1.36)	
Folate, *n*, %	26	8.1	46	9.9	20	14.8	78	12.8	85	13.9	18	22.0
Crude OR (95% CI)	1.00 (Reference)		1.25 (0.75–2.06)		**1.97 (1.06–3.67)**		1.00 (Reference)		1.10 (0.79–1.53)		**1.91 (1.08–3.39)**	
Adjusted OR (95% CI)	1.00 (Reference)		1.15 (0.69–1.93)		1.65 (0.87–3.16)		1.00 (Reference)		1.03 (0.74–1.45)		1.80 (1.00–3.27)	
Vitamin C, *n*, %	153	47.7	217	46.7	79	58.5	258	42.4	296	48.5	48	58.5
Crude OR (95% CI)	1.00 (Reference)		0.96 (0.72–1.28)		**1.55 (1.03–2.33)**		1.00 (Reference)		**1.28 (1.02–1.60)**		**1.92 (1.20–3.06)**	
Adjusted OR (95% CI)	1.00 (Reference)		0.87 (0.65–1.17)		1.25 (0.82–1.92)		1.00 (Reference)		1.20 (0.95–1.52)		**1.72 (1.05–2.80)**	
Minerals												
Calcium, *n*, %	189	58.9	267	57.4	91	67.4	327	53.8	364	59.7	52	63.4
Crude OR (95% CI)	1.00 (Reference)		0.94 (0.70–1.26)		1.44 (0.95–2.21)		1.00 (Reference)		**1.27 (1.01–1.60)**		1.49 (0.93–2.40)	
Adjusted OR (95% CI)	1.00 (Reference)		0.93 (0.69–1.26)		1.42 (0.91–2.20)		1.00 (Reference)		1.24 (0.98–1.56)		1.44 (0.88–2.35)	
Magnesium, *n*, %	156	48.6	225	48.4	76	56.3	222	36.5	263	43.1	34	41.5
Crude OR (95% CI)	1.00 (Reference)		0.99 (0.75–1.32)		1.36 (0.91–2.04)		1.00 (Reference)		**1.32 (1.05–1.66)**		1.23 (0.77–1.97)	
Adjusted OR (95% CI)	1.00 (Reference)		0.99 (0.74–1.33)		1.27 (0.83–1.93)		1.00 (Reference)		**1.31 (1.03–1.65)**		1.22 (0.75–1.98)	
Iron, *n*, %	23	7.2	26	5.6	19	14.1	237	39.0	270	44.3	44	53.7
Crude OR (95% CI)	1.00 (Reference)		0.77 (0.43–1.37)		**2.12 (1.11–4.04)**		1.00 (Reference)		1.24 (0.99–1.56)		**1.81 (1.14–2.88)**	
Adjusted OR (95% CI)	1.00 (Reference)		0.75 (0.41–1.36)		**2.03 (1.03–4.01)**		1.00 (Reference)		1.13 (0.84–1.51)		1.35 (0.74–2.46)	
Zinc, *n*, %	24	7.5	42	9.0	14	10.4	36	5.9	26	4.3	7	8.5
Crude OR (95% CI)	1.00 (Reference)		1.23 (0.73–2.07)		1.43 (0.72–2.86)		1.00 (Reference)		0.71 (0.42–1.19)		1.48 (0.64–3.45)	
Adjusted OR (95% CI)	1.00 (Reference)		1.24 (0.72–2.13)		1.57 (0.75–3.29)		1.00 (Reference)		0.63 (0.37–1.08)		1.22 (0.50–2.99)	
Copper, *n*, %	0	0	2	0.4	1	0.7	2	0.3	3	0.5	0	0
Crude OR (95% CI)	–		–		–		–		–		–	
Adjusted OR (95% CI)	–		–		–		–		–		–	
Nutrients with DG												
Protein, *n*, %	110	34.3	159	34.2	63	46.7	182	29.9	186	30.5	31	37.8
Crude OR (95% CI)	1.00 (Reference)		1.00 (0.74–1.35)		**1.68 (1.12–2.53)**		1.00 (Reference)		1.03 (0.80–1.31)		1.42 (0.88–2.30)	
Adjusted OR (95% CI)	1.00 (Reference)		0.97 (0.72–1.32)		**1.58 (1.03–2.40)**		1.00 (Reference)		1.00 (0.78–1.28)		1.30 (0.80–2.11)	
Fat, *n*, %	134	41.7	222	47.7	61	45.2	325	53.5	326	53.4	49	59.8
Crude OR (95% CI)	1.00 (Reference)		1.28 (0.96–1.70)		1.15 (0.77–1.73)		1.00 (Reference)		1.00 (0.80–1.25)		1.29 (0.81–2.07)	
Adjusted OR (95% CI)	1.00 (Reference)		1.28 (0.96–1.71)		1.12 (0.74–1.71)		1.00 (Reference)		1.00 (0.80–1.26)		1.31 (0.81–2.11)	
Saturated fat, *n*, %	121	37.7	200	43.0	59	43.7	338	55.6	361	59.2	46	56.1
Crude OR (95% CI)	1.00 (Reference)		1.25 (0.93–1.67)		1.28 (0.85–1.93)		1.00 (Reference)		1.16 (0.92–1.45)		1.02 (0.64–1.62)	
Adjusted OR (95% CI)	1.00 (Reference)		1.23 (0.91–1.65)		1.18 (0.77–1.80)		1.00 (Reference)		1.15 (0.91–1.44)		1.01 (0.63–1.61)	
Carbohydrate, *n*, %	121	37.7	167	35.9	43	31.9	219	36.0	204	33.4	32	39.0
Crude OR (95% CI)	1.00 (Reference)		0.93 (0.69–1.24)		0.70 (0.50–1.18)		1.00 (Reference)		0.89 (0.71–1.13)		1.14 (0.71–1.83)	
Adjusted OR (95% CI)	1.00 (Reference)		0.95 (0.70–1.28)		0.80 (0.52–1.25)		1.00 (Reference)		0.88 (0.69–1.11)		1.22 (0.75–1.98)	
Dietary fiber, *n*, %	211	65.7	319	68.6	107	79.3	399	65.6	440	72.1	60	73.2
Crude OR (95% CI)	1.00 (Reference)		1.14 (0.84–1.54)		**1.99 (1.24–3.21)**		1.00 (Reference)		**1.36 (1.06–1.73)**		1.43 (0.85–2.39)	
Adjusted OR (95% CI)	1.00 (Reference)		1.14 (0.83–1.56)		**1.91 (1.17–3.12)**		1.00 (Reference)		**1.32 (1.03–1.69)**		1.31 (0.77–2.23)	
Sodium (salt-equivalent), *n*, %	297	92.5	427	91.8	129	95.6	522	85.9	532	87.2	69	84.2
Crude OR (95% CI)	1.00 (Reference)		0.91 (0.53–1.55)		1.74 (0.69–4.35)		1.00 (Reference)		1.12 (0.81–1.56)		0.87 (0.46–1.65)	
Adjusted OR (95% CI)	1.00 (Reference)		0.95 (0.55–1.64)		1.88 (0.73–4.84)		1.00 (Reference)		1.11 (0.80–1.55)		0.86 (0.45–1.64)	
Potassium, *n*, %	202	62.9	305	65.6	107	79.3	348	57.2	383	62.8	49	59.8
Crude OR (95% CI)	1.00 (Reference)		1.12 (0.84–1.51)		**2.25 (1.40–3.62)**		1.00 (Reference)		1.26 (1.00–1.59)		0.88 (0.55–1.41)	
Adjusted OR (95% CI)	1.00 (Reference)		1.13 (0.83–1.53)		**2.17 (1.33–3.55)**		1.00 (Reference)		1.23 (0.97–1.56)		1.01 (0.62–1.65)	

*CI* confidence interval, *DG* tentative dietary goal for preventing lifestyle-related disease, *DRI* Dietary Reference Intakes, *EAR* estimated average requirement, *OR* odd ratio

Percentage of subjects whose intake was in the range of DG or above the EAR. Each energy-adjusted nutrient intake (units/day) was compared with each DRI value (units/day), using the cut-point method

The probability of inadequacy > 50% for menstruating women whose bioavailability of iron is 15% (< 9.3 mg/d) was considered inadequate for women aged 20–49 years

* Adjusted for confounding variables of age category (18–34, 35–50, and 51–64 years), occupation (professional / manager, sales / service / clerical, security / transportation / labour, student, housekeeper, and not in paid employment), living alone (yes or no), region (Hokkaido and Tohoku, Kanto, Hokuriku and Tokai, Kinki, Shikoku and Chugoku and Kyusyu), current smoker (yes or no) and habitual alcohol drinker (yes or no)

Table 4 shows energy-adjusted food group intakes according to the frequency of consuming meals prepared away from home. For both men and women, energy-adjusted vegetable intake in the Low groups was higher than that of the High group (*p* = 0.004 and *p* = 0.012 in men and women, respectively). Energy-adjusted fat and oil intake in the Low group was lower than the Moderate and High groups among men (*p* = 0.002); significant difference was observed only between the Low and Moderate groups in women. Among men, a higher intake of mushrooms adjusted for energy was observed in the Low group than in the High group (*p* = 0.015). Among women, a higher intake of potatoes and lower intake of meat and poultry adjusted for energy were observed in the Low group than in the Moderate group (*p* = 0.002 and *p* = 0.032, respectively).

**Table 4:** Energy-adjusted food group intake on the dietary recording day among 2221 Japanese adults aged 18–64 years according to frequency of consuming meals prepared away from home [Mean (SD)]†

Food groups (g/day)	Men					Women				
	Frequency of consuming meals prepared away from home			P^‡^	P^§^	Frequency of consuming meals prepared away from home			P^‡^	P^§^
	Low (*n =* 321)	Moderate (*n =* 465)	High (*n =* 135)			Low (*n =* 608)	Moderate (*n =* 610)	High (*n =* 82)		
Grains	637.8 (178.1)	636.2 (181.8)	668.0 (196.3)	0.187	0.372	431.9 (143.5)	434.7 (143.4)	468.6 (171.2)	0.099	0.148
Potatoes	66.5 (79.7)	56.8 (66.6)	52.7 (67.7)	0.086	0.063	59.3 (75.8)	44.5 (56.0)^*^	52.6 (74.1)	**0.001**	**0.002**
Sugars	7.9 (11.0)	7.1 (8.9)	7.7 (8.2)	0.507	0.694	7.1 (8.3)	7.1 (8.9)	5.5 (6.3)	0.226	0.292
Pulses	79.9 (101.3)	76.4 (83.3)	67.9 (92.8)	0.441	0.526	75.4 (91.6)	64.8 (74.7)	65.6 (90.3)	0.079	0.077
Sesame and nuts	2.2 (5.7)	3.3 (12.2)	1.7 (5.2)	0.119	0.202	2.4 (5.4)	2.8 (8.3)	1.9 (4.8)	0.378	0.376
Vegetables	354.3 (186.5)	362.4 (204.3)	294.2 (171.8)^*^	**0.001**	**0.004**	322.2 (171.6)	312.0 (161.7)	262.1 (160.8)^*^	**0.009**	**0.012**
Fruits	94.6 (149.3)	84.0 (114.6)	56.4 (125.3)	**0.016**	0.076	109.8 (120.0)	104.8 (130.6)	95.1 (123.3)	0.546	0.815
Mushrooms	22.9 (37.9)	18.6 (30.4)	13.1 (23.0)^*^	**0.010**	**0.015**	20.2 (28.7)	18.7 (30.3)	16.1 (25.7)	0.423	0.422
Seaweeds	15.5 (25.8)	12.0 (19.8)	11.9 (20.1)	0.068	0.086	11.4 (21.2)	10.1 (19.5)	11.0 (22.2)	0.541	0.593
Fish and shellfishes	88.5 (84.6)	87.7 (77.2)	71.7 (78.8)	0.091	0.276	67.7 (64.0)	66.1 (68.2)	67.1 (66.9)	0.913	0.856
Meat and poultry	131.1 (87.3)	134.8 (96.3)	138.2 (100.8)	0.739	0.988	91.5 (64.9)	101.1 (71.5)^*^	87.4 (66.4)	**0.027**	**0.032**
Eggs	48.8 (46.7)	45.4 (41.3)	43.3 (41.2)	0.391	0.419	41.4 (38.0)	39.3 (38.0)	38.0 (38.5)	0.536	0.597
Dairy products	100.9 (143.8)	104.3 (139.9)	97.0 (163.1)	0.863	0.744	124.5 (140.1)	124.5 (130.8)	124.0 (135.1)	0.999	0.992
Fat and oils	13.4 (10.2)	15.9 (11.0)^*^	17.5 (10.8)^*^	**< 0.001**	**0.002**	11.0 (9.0)	12.9 (9.5)^*^	13.2 (8.2)	**0.001**	**0.001**
Confectionaries	19.5 (42.0)	21.6 (43.2)	22.6 (54.1)	0.728	0.806	32.9 (47.6)	28.5 (44.3)	31.8 (40.5)	0.247	0.339
Beverages	1124.5 (684.1)	1146.0 (755.8)	1207.4 (748.5)	0.541	0.513	939.4 (544.3)	930.6 (542.6)	1000.7 (615.3)	0.554	0.491
Seasonings	115.5 (108.0)	135.7 (125.0)	147.4 (144.0)	**0.016**	0.084	84.9 (77.4)	93.6 (86.4)	97.4 (120.0)	0.139	0.125

^†^ Food groups expressed as amount per day were energy-adjusted by using the following equation: energy-adjusted intake (g/d) = observed intake (g/day) × EER (kcal/day)/observed energy intake (kcal/day)

^‡^ The *p* values are shown for an analysis of variance to analyze differences of nutrient intake between three groups

^§^ The *p* values are shown for covariate analysis to analyse difference of nutrient intake between three groups adjusted for confounding variables of age category (18–34, 35–50, and 51–64 years), occupation (professional / manager, sales / service / clerical, security / transportation / labour, student, housekeeper, and not in paid employment), living alone (yes or no), region (Hokkaido and Tohoku, Kanto, Hokuriku and Tokai, Kinki, Shikoku and Chugoku and Kyusyu), current smoker (yes or no) and habitual alcohol drinker (yes or no)

^*^ There is significant difference by Dunnett test compared with Low group in the adjusted model

The association between the frequency of eating out and take-away meals assessed through the one-day dietary record (classified into 3 groups according the frequency of consuming meals prepared away from home; never, once, and twice a day or more) and their nutrient intake inadequacy status is shown in the Supplementary Tables 1–4. Subjects in the “twice or more” group were less likely to have adequate vitamin and mineral intake, except vitamin B_1_, vitamin B_12,_ calcium and copper (both men and women), and vitamin B_2_ and vitamin C (only women) compared to the “never” group.

## Discussion

The present study examined the association between the frequency of consuming meals prepared away from home and nutrient intake inadequacy among Japanese adults aged 18–64 years. We found that inadequate intake of dietary fiber, vitamin C and several minerals was associated with a higher frequency of eating out or take-away meal. To the best of our knowledge, this study is the first to examine the association between the frequency of consuming meals prepared away from home and nutritional inadequacy, based on dietary reference values among Japanese adults.

In this study, participants were classified into three groups (Low, Moderate, and High group according to the frequency of consuming meals prepared away from home) based on the response to questions about the frequency of eating out and take-away meals. This categorization based on the questionnaire on the frequency of habitual eating out and take-away meals was comparable to that based on the dietary records, as shown in Table 1, despite being based on one-day dietary record method. Moreover, the association between the frequency of eating out and take-away meals assessed through the one-day dietary record and nutritional adequacy (Supplementary tables) showed similar results. A higher frequency of consuming meals prepared away from home was associated with less adequate dietary intake. Although the number of vitamins and minerals considered inadequate was larger when the classification using the dietary records was applied compared to that from the lifestyle questionnaire, the two different methods consistently supported our present findings.

Several factors increased the likelihood of eating food prepared away from home. Men and younger people had a higher frequency of consuming meals prepared away from home than others. This is consistent with previous studies that showed a higher frequency of eating out among men and younger adults when compared with that among older adults [15, 43], or higher proportion of eating out in men than women [15]. Similarly to a previous study among Japanese university students [37], living alone was associated with a higher frequency of eating meals prepared away from home. In addition, women living in urban areas had more eating out and take-away meals, which is consistent with the result of a previous study in Vietnam [44]. Thus, the current results may indicate that younger adults, especially men, are more likely to consume meals prepared away from home in Japan, as observed in other countries.

The association between the frequency of consuming meals prepared away from home and nutrients intake has been reported in several studies. Studies from Australia and Europe that used 24-h dietary recalls reported that adults with a higher frequency of consumption of foods prepared outside the home had lower intakes of iron and calcium, and vitamin C and calcium, respectively [2, 13]. Additionally, a review article reported that people with a higher frequency of eating out had lower vitamin C, iron and calcium intakes [12]. These reports showed similar results to the current results about energy-adjusted iron and calcium intakes. However, inadequate intake of these nutrients based on dietary reference values was not observed except for iron among men in the present study. According to a previous Japanese study, approximately more than 50% of Japanese adults had inadequate intake of calcium [45]. Also, another study showed that the proportion of Japanese women who met the standard value of iron intake was low, whereas a large percentage of Japanese men met the standard [46]. Japanese usual insufficient intake status may reflect to the present results, regardless of the frequency of consuming meals prepared away from home. In contrast, Japanese people rarely lack copper and protein [45], which may explain the current results where no difference was observed in the proportion of inadequate intake of these nutrients according to the frequency of consumption meals prepared away from home. Of note, EAR is set for the purpose of avoiding insufficient intake, whereas DG is set for the prevention of non-communicable diseases. It is possible that the definition of inadequacy of each nutrient intake may have determined differently, which suggests a caution of interpretation of the results.

Dietary fiber was the only nutrient that was observed in the inadequacy of intake depending on the frequency of consuming meals prepared away from home both in men and women. This finding is largely consistent with the previous studies that reported the association the frequency of eating out with dietary fiber intake [2, 8]. In this study, more inadequacy of dietary fiber intake was observed in the High group in men (OR (95%CI): 1.91 (1.17–3.12)), and the Moderate group in women (OR (95%CI): 1.32 (1.03–1.69)). It has been reported that women cook more often than men in Japan and other countries [42, 47]. The dietary fiber intake has been reported to be associated with frequency of cooking and cooking skill [48]. Higher income is associated with a higher frequency of eating out and take-away meals [41]. Additionally, better diets are seen in women compared with men [46], and highly educated individuals have greater dietary fiber and healthy food intake despite more frequent eating out and take-away [8]. These reports may partly explain our present findings that a higher frequency of consuming meals prepared away from home is associated with lower energy-adjusted nutrients intakes and inadequacy of nutrient intake compared with that in the Low group. Also, socioeconomic factor may be one of the important factors associated with consumption of meals prepared away from home. Unfortunately, other than occupation, we could not consider other socioeconomic indicators. While the proportion of occupation (professional, manager, sales, service, and clerics) differed among men and women, and was higher in the High group, there was no significant difference among the groups. Thus, future studies are needed with consideration of socioeconomic factors such as educational background and income level.

The frequency of eating out and take-away meals has been reported to be associated with a lower energy-adjusted intake of vegetable and a higher energy-adjusted intake of fat and oils [8, 18]. These results are consistent with the present study. Low intake of vegetables may partly explain the inadequate intake of potassium among men, and inadequate intake of magnesium and vitamin C among women. Especially, as there has been no report about inadequate intake of potassium and magnesium according to the frequency of consuming meals prepared away from home, our results may highlight the need for health promotion interventions for people with a higher frequency of eating out or take-away meals, as well as for the food industry.

In this study, approximately 45% of men and 30% of women regularly ate out or had take-away meal. In Japan, the government has called for voluntary efforts among the food industry to improve the food environment so that people can eat well-balanced meals, whether they eat out or prepare for themselves. Example of such efforts includes “increase in the number of corporations in the food industry that supply food products low in salt and fat.” [49] However, the current recommendation hugely focuses on preventing excess intake, and further efforts by the government are needed to increase the population intake of dietary fiber and minerals.

The study had some limitations. First, the participants were randomly selected from nationally representative households in Japan; however, the individual-level response rate was unknown. This might have introduced some bias in the estimation of average intake in Japanese adults. Also, the sample was essentially selected through a cluster of the households; however, it is difficult to treat them with the household cluster in our analysis. Second, a dietary intake derived from one-day weighed dietary record is unlikely to represent the usual intake. Therefore, the variability in the dietary intake of individuals over a period of several days might have influenced the findings. It is noted that the one-day household-based dietary record method used in NHNS has been compared with individual dietary records among Japanese participants, and the correlation coefficients of the intakes of total energy and macronutrients, such as protein, fat, and carbohydrates were high (0.89 to 0.91). Thus, this method may be valid for the estimation of individual intake [50]. Third, it could have been difficult for participants to accurately weigh food consumption in the case of eating out, take-away, or ready-meal use, unlike when they consumed home-cooked meals and could weigh all the foods and beverages, including the amounts of food waste and leftovers. Therefore, nutrient and food intakes may not have been accurately assessed. Fourth, we limited the participants to those who had three meals a day in the present analysis, because the purpose of this study was to assess nutrient intake and nutrition adequacy by the difference in the frequency of consuming meals prepared away from home. This might have induced some bias in the nutrient intakes. Fifth, we adjusted energy intake using EER assuming physical activity level to be level II for all participants due to the absence of quantitative information about physical activity, based on a previous study [51]. Therefore, it cannot be denied that this may have influenced the results of the current study. Finally, factors other than the frequency of consuming meals prepared away from home may also affect the adequacy of nutrient intake. Future studies should examine the causes of nutrient intake inadequacy.

## Conclusions

This cross-sectional study indicated that Japanese adults aged 18–64 years with a higher frequency of consuming meals prepared away from home were less likely to meet the standard values of dietary fiber, vitamin C and multiple minerals intake. Our findings suggest that these nutrients may be the focus of an interventional approach to improve the nutrient intake status of those with a high frequency of eating out and take away meals among Japanese adults. Further studies targeting food environment, including the food industry, are needed to improve nutritional adequacy for those with a higher frequency of eating out or consuming take-away meals.

## Supplementary Information

**Additional file 1.**

## Data Availability

This study was a secondary analysis of the 2015 National Health and Nutrition Survey in Japan and was conducted with the permission of the Ministry of Health, Labour and Welfare, in Japan.

## Abbreviations

ANCOVA: Covariate analysis
ANOVA: Analysis of variance
BMI: Body mass index
DG: Tentative dietary goal to prevent lifestyle-related diseases
DRIs: Dietary Reference Intakes for Japanese
EAR: Estimated average requirements
EER: Estimated energy requirement
NHNS: National Health and Nutrition Survey in Japan
OR: Odd ratio
95% CI: 95% confidence interval
